# Design-Challenges im virtuellen Raum – Ein Erfahrungsbericht und Handlungsempfehlungen

**DOI:** 10.1365/s40702-023-00977-x

**Published:** 2023-04-24

**Authors:** Marco Di Maria, David Walter, Thorsten Schoormann, Ralf Knackstedt

**Affiliations:** Institut für Betriebswirtschaft und Wirtschaftsinformatik, Universität Hildesheim, Universitätsplatz 1, 31141 Hildesheim, Deutschland

**Keywords:** Open Innovation, Innovationswettbewerb, Innovationswerkzeug, Innovationsplattform, Erfahrungsbericht, Design-Challenge, Konsortium., Open Innovation, Innovation Contest, Innovation Tool, Innovation Plattform, Experience Report, Design-Challenge, Consortium.

## Abstract

Die schnelllebige Entwicklung und Anwendung neuer digitaler Technologien führt zu einem erhöhten Innovationsdruck. Organisationen müssen sich an stetig verändernde Marktbedingungen anpassen und neue Ideen entwickeln, um Prozesse, Produkte und Geschäftsmodelle kontinuierlich zu erneuern. Für die Umsetzung solcher Ideen ist die Integration von Wissen über Disziplins- und Institutionsgrenzen hinaus gefordert. Da IT-gestützte Werkzeuge neue Möglichkeiten zur Unterstützung solcher Innovationsprozesse eröffnen, berichtet der vorliegende Beitrag über einen virtuellen Ideenwettbewerb – eine Design-Challenge – zum Thema ‚Einkaufserlebnis der Zukunft in Innenstädten‘. Dabei werden Erfahrungen aus der Vorbereitung und Durchführung synthetisiert. Der Wettbewerb wurde von einem Konsortium, bestehend aus einem universitären Forschungsprojekt, einem mittelständischen Unternehmen, einem regionalen Digitalisierungsinkubator, einer Großstadt und einem Start-Up, durchgeführt. Die teilnehmenden Teams bestanden aus Studierenden. Basierend auf den Erfahrungen und den kritischen Reflexionen aus der Vorbereitung und Durchführung des Wettbewerbs werden Handlungsempfehlungen für zukünftige virtuelle Design Challenges formuliert. Diese geben Unternehmen und öffentlichen Einrichtungen eine Orientierung hinsichtlich der Planung, Durchführung und Evaluation solcher Wettbewerbe.

## Abstract (en)

The rapid emergence and use of novel digital technologies lead to increased pressure to innovate. Organizations need to develop new ideas to adapt their processes, products and business models to changing market conditions. To implement those ideas, knowledge beyond disciplinary and institutional boundaries is necessary. As IT-supported tools open new opportunities to spur such innovation processes, this paper reports on a virtual idea competition (i.e., design challenge) concerned with the ‘future shopping experience in city centers’. Thereby, experiences from preparation and implementation are synthesized. The challenge was organized by a consortium consisting of a university research project, a medium-sized company, a regional digitalization incubator, a major city, and a start-up. Participating teams consisted of university students. Based on the collected experiences and critical reflection on the challenge’s preparation and execution, a set of recommendations for future virtual design challenges are formalized. These are intended to provide orientation for companies and public institutions concerning how to plan, perform, and evaluate such challenges.

## Chancen und Herausforderungen im Innovationsmanagement privater und öffentlicher Organisationen

Durch digitale Technologien, neue Regulatorik und gesellschaftliche Herausforderungen (Grand Challenges, Mertens und Barbian 2015) steigt der Innovationsdruck für Organisationen. Oft reicht es dann nicht mehr aus, ausschließlich auf altbewährtes Wissen und bestehende Strukturen zu setzen (Tsang und Zahra 2008). Organisationen müssen vielmehr eine Balance zwischen bestehenden und sicheren Ansätzen auf der einen Seite, sowie neuen, unklaren, aber potenzialträchtigen Prozessen, Produkten und Geschäftsmodellen auf der anderen Seite finden (O’Reilly und Tushman 2013). So stellt etwa im Rahmen von Smart Cities die Transformation öffentlicher Strukturen bei gleichzeitiger Bereitstellung notwendiger, laufender Dienste für Bürger:innen eine große Herausforderung dar (Leible et al. 2021). Da kreative Lösungen für das Innovieren unumgänglich sind (Brown 2008; Hofer et al. 2019), bedarf es der Etablierung eines integrierten, systematischen und kontinuierlichen *Innovationsmanagements*, das gleichermaßen die operative, taktische und strategische Ebene adressiert. So wird sowohl strukturell als auch prozessual die Innovationskultur gefördert und Organisationen können angemessen auf wechselnde Marktbedingungen reagieren. Setzen Organisationen, private Unternehmen oder Institutionen der öffentlichen Verwaltung (z. B. Gemeinden und Städte), dabei nicht nur auf eigene Ressourcen, sondern auch auf externe, wird von *offener Innovation *gesprochen.

Mit *organisationaler Ambidextrie* existiert ein strategischer Ansatz, der es ermöglicht, einen Ausgleich zwischen den beiden widerstrebenden Polen der „Exploitation“ und „Exploration“ zu schaffen. Während sich Exploitation auf das Ausnutzen bestehender Ressourcen und Kompetenzen bezieht, forciert Exploration das Ausprobieren neuer Wege mit neuen Mitteln, unabhängig von deren Erfolgsaussichten (March 1991). Bei der Umsetzung organisationaler Ambidextrie müssen Organisationen verschiedene Entscheidungen treffen. Eine Entscheidung betrifft die strukturelle Ambidextrie in Bezug auf die Organisationsstrategie (Saxena 2020). Dabei wird festgelegt, inwiefern neben bestehenden Organisationseinheiten neue, separate Strukturen geschaffen werden sollen, in denen Mitarbeiter:innen nach anderen Regeln (z. B. Agilität) arbeiten. Organisationen müssen zudem entscheiden, bis zu welchem Grad sie Aktivitäten mit eigenen Mitteln durchführen und/oder auf externe Ressourcen (z. B. Wissen und Infrastruktur) setzen möchten (Hwang et al. 2021).

Eine *Design-Challenge* ist ein Innovationswettbewerb in einem *Open-Innovation-Format *zur Umsetzung von organisationaler Ambidextrie. Das Format ermöglicht die Bearbeitung einer gemeinsamen Fragestellung bei gleichzeitiger Erreichung individueller Ziele. Teilnehmer:innen bearbeiten individuell oder in Gruppen mit Hilfe interner und externer Ressourcen eine konkrete Fragestellung. Diese wird von einer veranstaltenden Organisation definiert, die entsprechendes Interesse an einer Lösung mitbringt. Veranstalter:innen können zentrale Parameter flexibel ausgestalten, u. a. das Durchführungsmedium (online, offline, gemischt). Vor diesem Hintergrund bleibt zu kären, wie Design-Challenges in heterogenen Konsortien konzipiert werden können. Der vorliegende Beitrag berichtet über die Planung und Durchführung einer solchen virtuellen Design-Challenge, die ein institutionsübergreifendes Konsortium veranstaltete. Das Konsortium bestand aus einer Großstadt, einem Digitalisierungsinkubator, einem Forschungsprojekt sowie einem Startup. Ziel der teilnehmenden studentischen Teams war es, kreative Ideen und Lösungsansätze für Fragestellungen im Kontext ‚Einkaufserlebnis der Zukunft in Innenstädten‘ zu entwickeln. Die Umsetzung der Design-Challenge erfolgte vollständig digital, um Potenziale und Herausforderungen – insbesondere durch die Konsortialstruktur – bei dieser Art der Umsetzung zu untersuchen. Basierend auf den Ergebnissen und gesammelten Erfahrungen werden im Folgenden Handlungsempfehlungen abgeleitet, die sowohl für interessierte Praktiker:innen als auch Innovationsforscher:innen nützlich sind.

Der Beitrag geht zunächst auf Open-Innovation-Konzepte ein und erläutert dann die Struktur der bundesweiten, virtuellen Design-Challenge mit Studierenden. Anschließend werden Best Practices aus der Sicht sämtlicher Konsortial-Partner:innen herausgearbeitet, die Unternehmen und öffentlichen Einrichtungen eine Orientierung hinsichtlich der virtuellen Durchführung von Innovationsprozessen bieten. Insgesamt wird damit ein wichtiger Beitrag zur Förderung von Ideen als Reaktion auf sich verändernde Situationen geleistet.

## Virtuelle Innovationswettbewerbe für offene Innovation

In einem* Innovationsökosystem *kommen verschiedene Organisationen, z. B. aus der Wissenschaft, Verwaltung und Privatwirtschaft, zusammen, um ein übergeordnetes Ziel zu verfolgen (Smith et al. 2016). Im Zentrum steht meist ein gemeinsames Produkt, das von einem Konsortium verwaltet und durch weitere Akteur:innen unterstützt wird (Adner und Kapoor 2010). Da für den Erfolg eines Innovationssystems die Interessen der Beteiligten stets ausgeglichen sein müssen, sind die Aspekte Kommunikation, Koordination und Kollaboration essentiell (z. B. Österle und Otto 2010; Kammler et al. 2020). Diese Ökosysteme realisieren den Ansatz der *offenen Innovation *(Enkel et al. 2009)*. *Im Gegensatz zu geschlossener Innovation, bei der ausschließlich eigene Ressourcen genutzt werden und meist keine organisationsübergreifenden Kollaborationen stattfinden, setzt die offene Innovation systematisch interne und externe Ressourcen zur Erreichung eigener und gemeinschaftlicher Innovationsziele ein (Raisch et al. 2009). So kann etwa eine involvierte Organisation ihren Kooperationspartner:innen neue Technologien oder Werbekanäle zur Verfügung stellen. Dies erlaubt ein risikoarmes und ressourceneffizientes Erproben neuartiger Strategien im Ökosystem (*Exploration*) bei gleichzeitiger Sicherstellung der Effizienz des Kerngeschäfts (*Exploitation*) (March 1991). So kann strukturelle Ambidexterität erreicht werden (Kusanke et al. 2022). Organisationen beginnen zunächst mit kleineren Organisationseinheiten (z. B. einzelnen Teams) und können mit zunehmenden Erfahrungen auch größere Teile (z. B. mehrere Teams, weitere IT-Infrastruktur) in das Innovationsökosystem einbringen (March 1991; Saxena 2020).

*Innovationswettbewerbe *zählen zu den bewährten Ansätzen zur Realisierung offener Innovation in größeren Ökosystemen und Verbünden (Haller et al. 2011). Es sind Wettbewerbe, bei denen Teilnehmer:innen ihre Fähigkeiten, Erfahrungen und ihr kreatives Potenzial einsetzen, um eine Lösung für eine von einer Organisation vorgegebenen Fragestellung zu entwickeln (Bullinger und Möslein 2010). Durch die Kombination von Ressourcen der verschiedenen Beteiligten für einen begrenzten Zeitraum können gemeinsame Ziele erreicht und komplexe Aufgaben gelöst werden. Sie gelten als sog. „knowledge broker“ (Smith et al. 2016, S. 3), die Wissen zwischen verschiedenen Organisationen vermitteln und den Austausch von Wissen und Ressourcen erleichtern, um den Innovationsprozess zu fördern. Dabei können z. B. digitale Produkte oder Dienstleistungen in Form von Ideen oder Prototypen generiert werden. In Bezug auf die strategische Zielsetzung lassen sich gesellschaftliche und unternehmerische Wettbewerbe unterscheiden (Haller et al. 2011): *Gesellschaftlich-orientierte Wettbewerbe* sprechen eine breite Gruppe potenzieller Teilnehmer:innen an und stellen Aspekte wie technologischen Wissensfortschritt, Lösung gesamtgesellschaftlicher Probleme (z. B. Nachhaltigkeit), Förderung von Kompetenzentwicklung oder Standortentwicklung in den Vordergrund. *Unternehmerische Wettbewerbe* setzen eher frühzeitige Trenderkennung, Ko-Kreation, Branding und Rekrutierung in den Fokus. Eine weitere Unterscheidung betrifft die Richtung des Wissenstransfers (Enkel et al. 2009). Ein Innovationsökosystem kann sich entweder *outside-in* öffnen (d. h. von außen Ressourcen ins eigene Netzwerk lassen) oder *inside-out* öffnen (d. h. von innen Ressourcen aus dem Netzwerk herauslassen). *Coupled* beschreibt eine Kombination beider Arten.

Eine besondere Form dieser Wettbewerbe sind Design-Challenges, bei denen Teilnehmer:innen kreative Lösungen für ein spezifisches Designproblem entwickeln. Typischerweise geht es um die Entwicklung ganzheitlicher, innovativer Lösungen für Probleme bestimmter Zielgruppen, die sowohl funktional als auch ästhetisch ansprechend sein sollen. Sie sind eine effektive Möglichkeit, um im Rahmen offener Innovation Ideen und Lösungen für komplexe Probleme zu generieren und somit den Innovationsprozess zu fördern.

## Design-Challenge: Einkaufserlebnis der Zukunft in Innenstädten

In diesem Kapitel erläutern wir zunächst die übergeordnete Struktur und Zielsetzung der Design-Challenge. Anschließend beschreiben wir den konkreten Aufbau und Ablauf der Challenge unter Verwendung des konzeptionellen Rahmens zur Klassifikation von Innovationswettbewerben von Bullinger und Möslein (2010).

### Struktur und Ziele der Design-Challenge

**Abb. 1:**
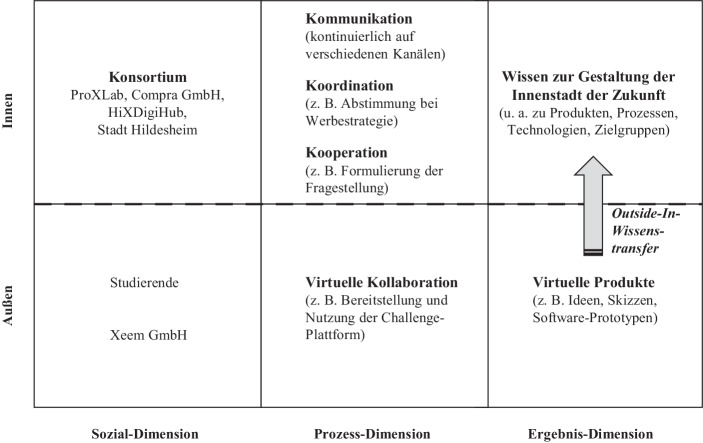
Innovationsköksoystem für die Design-Challenge ‚Einkaufserlebnis der Zukunft in Innenstädten‘

Das Ziel der Design-Challenge ‚Einkaufserlebnis der Zukunft in Innenstädten‘^1^ bestand in der kundenzentrierten Ideenentwicklung durch studentische Teams zur Verbesserung des Einkaufserlebnisses in der Innenstadt. Die Planung erfolgte durch ein niedersächsisches Konsortium, das von einem Startup für Innovationsplattformen unterstützt wurde. Das Konsortium wirkte als temporäres Innovationsökosystem, das sich zeitlich befristet zusammengeschlossen hat, um mittels offener Innovation eine gesellschaftlich-relevante Fragestellung zu bearbeiten. Für die Umsetzung der Design-Challenge wurden diverse Personen und Institutionen eingebunden, die durch ihr heterogenes Profil vielfältige Kompetenzen und Ressourcen einbrachten (siehe Abb. 1).

Das universitäre *Forschungsprojekt ProXLab*^2^ hat die Bildung des Konsortiums initiiert. Sein Ziel war es herauszufinden, inwiefern sich Innovationswettbewerbe als kooperatives Lehrformat eignen (Kammler et al. 2020), um Studierenden frühzeitig den Aufbau praxisrelevanter Kompetenzen in echten Problemsituationen ermöglichen zu können (Goldkuhl et al. 2017). Die* Stadt Hildesheim* hatte Interesse an der Lösung eines gesellschaftlich-relevanten Problems. Als Teil der Smart City-Initiative fördert sie den Erfahrungsaustausch und Wissensaufbau mit virtuellen Beteiligungsformaten – hier für die Gestaltung der Innenstadt der Zukunft. Das mittelständische *Unternehmen Compra GmbH* aus dem Bereich E‑Commerce strebte die Erprobung eines offenen Innovationsmanagements an, ohne die eigene (IT-)Organisation und damit verbundene Prozesse verändern zu müssen. Zudem fungiert der Innovationswettbewerb als Rekrutierungswerkzeug. Der *Digitalisierungsinkubator HiXDigiHub* wollte neue digitale Innovationsformate im regionalen Kontext erproben. Das *Startup Xeem GmbH *erprobte sein Geschäftsmodell in universitären und gesellschaftlichen Anwendungen.

### Aufbau und Ablauf der Design-Challenge

**Abb. 2:**
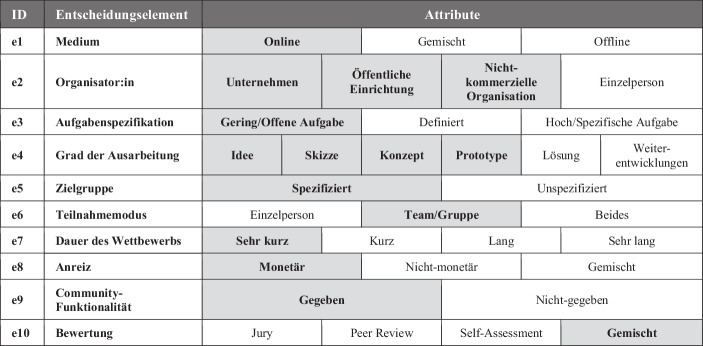
Ausprägungen der Design-Challenge

Bei der Ausgestaltung eines Innovationswettbewerbs gilt es, diverse Entscheidungen zu treffen. Mit einem geeigneten Planungsrahmen wird eine effiziente Vorbereitung und Durchführung des Innovationswettbewerbs möglich (Smith et al. 2016). Hierfür bieten Bullinger und Möslein (2010) zehn Elemente (siehe Abb. 2). Nachfolgend erläutern wir die konkreten Ausprägungen (siehe grau hinterlegte Begriffe) im Rahmen der Design-Challenge für ‚Einkaufserlebnis der Zukunft in Innenstädten‘, die im Konsortium festgelegt wurden.

Die durchgeführte Design-Challenge stellt einen vollständig virtuellen (e1) Innovationswettbewerb dar, der von mehreren Organisationen (e2) veranstaltet wurde. Ursprünglich war eine hybride Durchführung mit Präsenzveranstaltungen geplant, was aufgrund von Kontaktbeschränkungen bedingt durch die Covid-19-Pandemie nicht möglich war. Die offene (e3) Fragestellung lautete: *„Wie müsste das Shopping Erlebnis aus Kundensicht sein, damit lokales Einkaufen wieder eine attraktive Alternative neben dem Online-Shopping wird“.* Diese Formulierung sollte studentischen Teams (e5, e6) diverse Lösungsansätze (e4) ermöglichen. Den Teams wurden Hinweise zur Lösungserstellung in der kollaborativen Arbeitsumgebung auf der Challenge-Plattform bereitgestellt. Dazu zählten Leitfragen und Design-Thinking-Methoden (z. B. Customer Journey Map oder Personas). Die Bearbeitungszeit der Design-Challenge war mit zwei Stunden sehr kurz (e7). Die Bearbeitungsphase wurde durch einen virtuellen Kick-Off, eine abschließende Pitch-Session und Live-Voting ergänzt. Insgesamt haben fünf studentische Teams teilgenommen. Für den Hauptpreis wurden die Abgaben der Teams von einer Jury, bestehend aus Vertreter:innen des Konsortiums, auf Basis der eingereichten Lösungsidee ausgewählt. Zu gewinnen gab es für den 1. Platz ein Preisgeld von 700 € und für den 2. Platz 300 €, gesponsert durch die Compra GmbH sowie unterstützt durch die Stadt Hildesheim und ProXLab. Zusätzlich zur eigentlichen Challenge wurde bei der Abschlussveranstaltung ein Pitch-Contest mit einem Live-Voting^3^ durchgeführt, bei dem jedes Team seine Idee vorstellen konnte. Anschließend konnte jede am Event teilnehmende Person abstimmen, um den Pitch-Winner zu ermitteln. Für das siegreiche Team beim Live-Pitch wurde ein Gutschein im Wert von 20 € vorgesehen. Es bestanden also in zweifacher Hinsicht monetäre Anreize (e8). Des Weiteren stand jedem Team ein eigener, virtueller Arbeitsraum auf der Challenge-Plattform zur Verfügung, in dem sich die Team-Mitglieder über die Fragestellung austauschen konnten (e9). Die Bewertung der eingereichten Ideen erfolgte schließlich auf gemischte Weise mittels Jury und Peer Reviews (e10).

### Vorbereitung und Durchführung der Design-Challenge

**Abb. 3:**
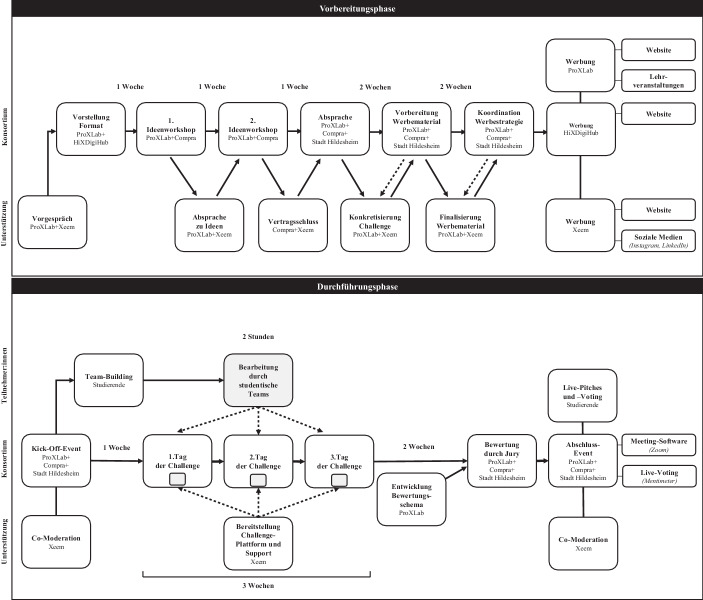
Ablauf der Design-Challenge ‚Einkaufserlebnis der Zukunft in Innenstädten‘

Für die Beschreibung des tatsächlichen Ablaufs der Design-Challenge werden die Phasen der Vorbereitung und Durchführung unterschieden, die von Einzel- und Kollaborationsaktivitäten geprägt waren (siehe Abb. 3).

In der *Vorbereitungsphase* wurden zentrale Aufgaben für eine erfolgreiche Durchführung der Design-Challenge geklärt (siehe Abb. 3). Zuerst wurde die grundlegende Idee einer Challenge zwischen *ProXLab* und der *Xeem GmbH* erörtert und geplant. Das Vorhaben wurde bei einer Veranstaltung des *HiXDigiHub* vorgestellt, die von kleinen und mittelständischen Unternehmen der Region sowie öffentlichen Institutionen besucht wurde. Danach entschieden der *HiXDigiHub*, die *Compra GmbH* und das *Smart-City-Projekt der Stadt Hildesheim* mitzuwirken. Es wurden mehrere Workshops durchgeführt, um die Fragestellung und den organisatorischen Rahmen zu bestimmen. Zunächst wurden drei bis fünf praxisrelevante Fragestellungen mit der *Compra GmbH *entwickelt, die sich im Austausch mit der Smart-City-Initiative befand. Hierfür wurde eine visuell unterstützte Variante der Think-Aloud-Methode mit einer virtuellen Whiteboard-Lösung (hier MIRO) verwendet. Dabei tätigte ein Vertreter der *Compra GmbH* Äußerungen, die ihm digital in komprimierter Form gespiegelt wurden. So wurde die iterative Ideenentwicklung unterstützt und es konnte schnell eine Ausarbeitung einzelner Ideen erfolgen. Zur Sicherstellung der Durchführbarkeit der Ideen tauschten sich das *ProXLab* und die *Xeem GmbH* regelmäßig aus. Mit den Workshops konnte die konkrete Challenge und der dazugehörige Rahmen definiert werden, z. B. Anzahl der Teams, Dauer, methodische Hilfen (siehe Abschn. 3.2). Damit wurde die Basis für eine vertraglich-bindende Zusammenarbeit zwischen der *Compra GmbH* und der *Xeem GmbH* geschaffen.

Die *Durchführungsphase* begann mit einem Kick-Off am Digitaltag 2021 (siehe Abb. 3). Interessierte Personen konnten sich direkt bei Ansprechpartner:innen aus dem Konsortium über die Challenge informieren. Nachdem sich Studierende in Teams zusammengefunden hatten, konnten sie sich auf der Challenge-Plattform registrieren. Jedes Team konnte für zwei Stunden auf der Plattform seine Idee entwickeln, wobei der Bearbeitungsbeginn frei gewählt werden konnte. Die Bearbeitung musste jedoch bis zum Ende des dritten Tages der Challenge abgeschlossen sein. An drei Tagen wurde zusätzliche Unterstützung per E‑Mail angeboten, falls technische, organisatorische oder methodische Probleme auftraten. Die Zusammenarbeit erfolgte in einem Live-Text-Editor und das Ergebnis konnte als PDF hochgeladen werden. Nach Abgabe bewertete eine Jury die eingereichten Ideen. Dafür wurde von *ProXLab* ein individuelles Bewertungsschema^4^ entwickelt, das eine vergleichbare Grundlage zur Beurteilung schaffen sollte. Erwähnenswert ist hier das Vorgehen der *Compra GmbH*: Die Bewertung des Unternehmens wurde nicht einfach vom Geschäftsführer vorgenommen, sondern mittels bottom-up-Ansatz ermittelt. Die Mitarbeiter:innen durften abstimmen und es wurde ein Durchschnitt aller Bewertungen ermittelt. Da die Mitarbeiter:innen überwiegend aus Hildesheimer Bürger:innen bestehen, zeigten sie großes Interesse an der Design-Challenge. Nach Konsolidierung der Bewertungen wurden die Platzierungen ermittelt und das Ergebnis im Konsortium geteilt. Beim Abschlussevent erfolgten zudem „Short Pitches“, bei denen jedes Team die eigene Idee in maximal fünf Minuten vorstellte. Über diese wurde per Live-Voting durch alle Event-Teilnehmer:innen abgestimmt. Damit wurde neben dem Hauptpreis ein weiterer Anreiz zur Eventteilnahme geschaffen. Dann wurden die siegreichen Teams prämiert. Der erste Platz ging an ein Team, das ein Mockup für eine mehrsprachige Shopping-App entwickelt hat, die Einkaufen, Mobilität und weitere Bedürfnisse integriert bereitstellt. Der zweite Platz ging außerplanmäßig an zwei Teams, da sich die Jury bei der Bewertung nicht zweifelsfrei entscheiden konnte. Die Ideen dieser zweitplatzierten Teams umfassten exotische Essensstände, Locations für Selfies und Teilen in sozialen Medien, besondere Events, interaktive Spiegel, robotische Dienstleistungen für Transport und Beratung, sowie Mini-Challenges in der Stadt. Zum Abschluss wurde ein offenes Gespräch unter allen Teilnehmer:innen initiiert, um einen gemeinsamen Erfahrungsaustausch zu ermöglichen. Dabei wurden sowohl positive als auch negative Aspekte herausgearbeitet.

Die Teilnehmer:innen lobten den starken Praxisbezug der Challenge als Ergänzung zu den Veranstaltungen in der universitären Lehre. Besonders positiv äußerten sich die Teams, die aus Studierenden verschiedener Studiengänge bestanden. Sie konnten mehrere Perspektiven zur Problemlösung einsetzen. Die *Compra GmbH* und die *Stadt Hildesheim* zeigten sich ebenso positiv und betonten, dass das Format interessant und u. a. nützlich für Challenges mit Bürger:innen sei. Dabei könnten gemischte Teams aus Studierenden, Schüler:innen, Auszubildenden, Fachkräften, Rentner:innen und Vertreter:innen weiterer Gruppen zusätzliche spannende Perspektiven beitragen. Kritische Äußerungen gab es zur Bearbeitungszeit. Die Ideenentwicklung und Nutzung der bereitgestellten Methoden in nur zwei Stunden wurde als herausfordernd empfunden. Ein längerer Zeitraum wäre wünschenswert, sodass Studierende besser Teams bilden und sich mit den Hilfsmaterialien und der Plattform intensiver auseinandersetzen können. Auch die offene Fragestellung wurde als schwierig empfunden, da sie einen großen Interpretationsspielraum bot und so einige Teilnehmer:innen verunsichert hat. Sie waren sich nicht sicher, was genau erwartet wurde. Ein erweitertes Unterstützungsangebot in Form von Feedback wurde gewünscht, um besser zu verstehen, wie die eigenen Kompetenzen, u. a. virtuelle Kollaboration, Problemlösung, Design Thinking, noch stärker entwickelt werden können. Aus dem Konsortium gab es kritische Bemerkungen zur Umsetzung, die auf mangelnde praktische Erfahrung der Partner:innen mit der Durchführung von Design-Challenges im virtuellen Raum zurückgeführt wurde. Die *Stadt Hildesheim* erwähnte, dass die Lösungen stark auf Studierende fokussiert waren und eher weniger die Bedürfnisse einer diversen Bürgerschaft adressierten.

## Kritische Reflexion und Handlungsempfehlungen

Innovationswettbewerbe können ein effektives Werkzeug zur Umsetzung offener Innovationstrategien sein, sind jedoch mit Herausforderungen verbunden. Basierend auf der Reflexion der praktischen Umsetzung eines solchen Innovationswettbewerbs – von der Vorbereitung, über die Durchführung bis hin zum Ergebnis – werden daher nachfolgend Handlungsempfehlungen für die einzelnen Phasen abgeleitet und diskutiert.

### Phase: Vorbereitung

Für die Vorbereitungsphase der Design-Challenge haben wir basierend auf unseren Beobachtungen und Diskussionen während und nach dem Wettbewerb fünf Hauptaspekte identifiziert. Hierfür haben wir fünf Handlungsempfehlungen (V1–V5) formuliert.

#### Klärung der Organisation und deren Konsequenzen (V1)

Zu Beginn sollte klar sein, wer die Challenge organisiert, d. h. eine Organisation allein oder ein Konsortium. Letzteres erhöht die Komplexität und ist mit großem Aufwand für Kommunikation, Koordination und Kollaboration verbunden. Andererseits stehen einem Konsortium mehr Ressourcen zur Verfügung. Daher haben wir unseren Innovationswettbewerb im Konsortium organisiert. Das war nicht immer einfach und es konnten nicht alle Ziele der Konsortial-Partner:innen erreicht werden. Beispielsweise wollte das *ProXLab* untersuchen, inwiefern Studierende durch die Teilnahme an der Challenge kompetenter in Bezug auf virtuelle Kollaboration und Design-Thinking werden. Das war letztlich nicht vollständig abbildbar. Eine einfachere Organisationsstruktur wäre hier eventuell sinnvoller gewesen, um die Komplexität der Organisation gering zu halten und so das Untersuchungsziel zu erreichen.

#### Etablierung einer transparenten Kommunikation (V2)

Jede beteiligte Partei sollte verbindlich Personen benennen, die als Ansprechpartner:innen im Kontext der Challenge bereitstehen. Gewünschte Kommunikationswege und Anwendungen sollten bereits in den frühen Phasen festgelegt werden. Probleme können so frühzeitig erkannt und gemeinsam gelöst werden. In unserem Fall war z. B. die *Compra GmbH* aufgrund mangelnder Erfahrung mit der Umsetzung von Challenges unsicher und konnte daher nicht abschätzen, inwieweit die* Xeem GmbH *die Erwartungen erfüllen kann. Da das Problem sehr offen kommuniziert wurde und die *Xeem GmbH* bereits Erfahrungen mit vergangenen Challenges einfließen lassen konnte, wurden Zweifel schnell beseitigt.

#### Entgegenbringung von Vertrauen (V3)

Angesichts vieler Kommunikationsschleifen, Verständnisschwierigkeiten und etwaigen kulturellen Unterschieden zwischen Organisationen ist ein offenes, tolerantes und flexibles Mindset zu empfehlen. Gerade wenn es aufgrund häufiger bilateraler Austausche zu temporären Informationsdefiziten kommt, ist Geduld von Vorteil. Im vorliegenden Fall stellte z. B. *Xeems* agile Arbeitsweise mit vielen Entscheidungen ohne wochenlange Planung zunächst eine Hürde für *ProXLab* dar. Nach und nach passten sich beide Seiten an und es entstand eine vertrauliche und produktive Zusammenarbeit.

#### Planung und Durchführung als gemeinsame Lernerfahrung (V4)

Aufgrund der vielen Interdependenzen der teils komplexen Planung und Durchführung sollte mit unerwarteten Ereignissen geplant werden. Es sollte dann gemeinsam am Verständnis und der Lösung des Problems gearbeitet werden. In unserem Fall war die Zusammenarbeit mit städtischen und universitären Institutionen für die *Xeem GmbH* neu und einige Prozesse verliefen nicht so wie bei vorherigen Challenges. Teilweise stellte die im Konsortium verteilte Kontrolle und Steuerung ein Problem dar. Im Verlauf der Kooperation ergaben sich dadurch jedoch Chancen. So konnte durch verteilte Steuerung die Effizienz in der Zusammenarbeit sogar gesteigert werden.

#### Berücksichtigung einer effektiven Rekrutierung von Teilnehmer:innen (V5)

In der Vorbereitung sollte sichergestellt werden, dass genügend Personen teilnehmen und diese aus der gewünschten Zielgruppe rekrutiert werden. Hierfür sollte ausreichend Zeit eingeplant werden, um geeignete Kommunikationskanäle auszuwählen. Bei unserer Challenge haben wir die bereits bestehenden Kanäle auf sozialen Medien der *Xeem GmbH* sowie Direktansprache in Lehrveranstaltungen durch universitäre Vertreter:innen genutzt. Wir haben etwa zwei Monate vor Challenge-Beginn mit der Werbung begonnen, um Studierende als Teilnehmer:innen zu rekrutieren. Zunächst haben wir in zweiwöchentlichen Abständen geworben und dann nochmals intensiv täglich in den letzten 14 Tagen vor dem Kick-Off-Event. Das stellte sich als zielführend heraus und wir konnten in ausreichendem Maße Studierende zur Teilnahme motivieren.

### Phase: Durchführung

Für die Phase der Durchführung virtueller Design-Challenges haben wir drei wesentliche Aspekte als zentral bestimmt und drei Handlungsempfehlungen (D1–D3) abgeleitet.

#### Angebot an begleitenden Veranstaltungen (D1)

Wir empfehlen, die Teilnehmer:innen zu jedem Zeitpunkt ausreichend über Ablauf, Inhalt und Erwartungen der Challenge zu informieren. So wird nicht nur die Wahrscheinlichkeit eines besseren Ergebnisses erhöht, sondern in gewissem Maße auch die Motivation der Teilnehmer:innen. Wir haben bei unserer Challenge gute Erfahrungen mit einem Kick-Off zu Beginn und einem Abschlussevent gemacht. Die Teilnehmer:innen erachteten insbesondere die Erläuterung der Challenge und die damit verbundenen Erwartungen direkt durch die veranstaltenden Personen aus dem Konsortium als sehr gut.

#### Angemessene Auswahl des Bearbeitungszeitraums (D2)

Die Dauer der Challenge sollte auf die Fragestellung sowie auf die Fähigkeiten und Erfahrungen der Teilnehmer:innen abgestimmt sein. In unserem Fall war die Dauer mit zwei Stunden reiner Bearbeitungszeit ohne Möglichkeit zur Unterbrechung sehr kurz. Ein längerer Bearbeitungszeitraum hätte Teilnehmer:innen helfen können, sich noch besser auf der Plattform zurechtzufinden und letztlich im Team produktiver zusammenzuarbeiten.

#### Implementierung von Coaching-Elementen (D3)

Es könnte Coaching eingesetzt werden, damit sich die Teams auf die Bearbeitung der Fragestellung konzentrieren können und dabei ihr Ziel nicht aus den Augen verlieren. So könnten Coaches bei der Nutzung der technischen Infrastruktur begleiten, beim Einsatz von (Design‑)Methoden unterstützen oder bei der Erreichung von Kompetenzzielen helfen. Vor allem bei bildungsorientierten Challenges sehen wir hier aus Sicht von Hochschuldozierenden noch weiteres Potenzial. Aus Sicht der *Xeem GmbH* waren diese Aspekte zum Zeitpunkt der Challenge nur schwer umzusetzen, da das Geschäftsmodell und die Plattform bis dato nur auf unternehmerische Challenges ausgerichtet waren, die weniger die methodisch-fundierte Kompetenzentwickung im Blick hatten. Die *Xeem GmbH* nahm das als Anlass zur Anpassung der Challenge-Plattform, um künftig kompetenzorientierte (Lern‑)Challenges im Bildungskontext zu ermöglichen.

### Phase: Ergebnis

Wir haben Herausforderungen hinsichtlich der Erwartungen an die von den Teams kreierten Ergebnissen extrahiert. Hierfür haben wir diese drei Handlungsempfehlungen (E1–E3) formuliert:

#### Kommunikation der Art und des Umfangs des Ergebnisses (E1)

Aufgrund des Zeitdrucks sollten sich Teilnehmer:innen schnell vergegenwärtigen, welche Leistung sie erbringen sollen. Zur Ideengenerierung in einem offenen Innovationsprozess ist ein Wettbewerb ein geeignetes Werkzeug. Falls jedoch beispielsweise eine komplexe Softwareanwendung als Ergebnis in einer kurzen Design-Challenge erwartet wird, könnten andere Formate geeigneter sein. In unserem Fall wurden nur wenige konkrete Anforderungen an eine Lösung vorab definiert und kommuniziert. So lieferten die Studierenden vielfältige Lösungsarten, u. a. textuelle Beschreibungen, visuelle Konzepte und App-Prototypen, die durch Personas, Moodboards und Skizzen unterstützt wurden. Die Ausarbeitungsgrade variierten jedoch stark zwischen den Gruppen. Wir empfehlen daher eine frühzeitige Klärung der eigenen Erwartungen und eine eindeutige Kommunikation des erwarteten Ergebnisses an potenzielle Teilnehmer:innen.

#### Festlegung des Abgabeformats (E2)

Das Spektrum möglicher Abgabeformate (z. B. Text, Audio, Bild, Video) ist frühzeitig zu bestimmen. Der Lösungsraum sollte nicht zu stark eingeschränkt sein, sodass das Ideenpotenzial der Teams gut ausgeschöpft werden kann. Gleichzeitig sollte er auch nicht zu breit sein, um eine Vergleichbarkeit bei der Bewertung sicherzustellen. In unserem Fall war nur eine Abgabe im PDF-Format möglich, was einige Teams als Hindernis für die eigene Ideenentfaltung empfanden, die Bewertung aber vereinfachte. Ausgehend vom eingesetzten Bewertungsschema und Weiterverwendung der Ergebnisse, sollte hier zunächst ein Konsens im Konsortium gefunden und dann ein geeignetes Format festgelegt werden.

#### Abgleich des (angestrebten) Ergebnisses mit dem Profil der Teilnehmer:innen (E3)

Vorab ist es schwer einschätzbar, welche Fähigkeiten und Erfahrungen die Teilnehmer:innen einbringen. In unserem Fall bestanden die Teams aus Studierenden, deren Lösungen stark auf Studierende abzielten. Durch heterogenere Teams könnte dem entgegengewirkt werden. Beispielsweise könnten gemischte Teams aus Studierenden, Fachkräften aus Unternehmen oder öffentlicher Verwaltung sowie Vertreter:innen aus Zielgruppen, für die designt wird, gebildet werden. So könnte die Wahrscheinlichkeit einer balancierten Mischung aus fachlicher Expertise, Methodenwissen und Erfahrung gesteigert werden.

### Phase: Unterstützung

Letztendlich haben wir über die gesamte Design-Challenge hinweg Beobachtungen hinsichtlich der Unterstützung von (digitalen) Werkzeugen und Methoden gemacht. Auf deren Basis haben wir die folgenden zwei Empfehlungen (U1–U2) ausgearbeitet:

#### Bereitstellung von Werkzeugen zur kreativen Kollaboration (U1)

Der Prozess der gemeinsamen Ideenentwicklung im virtuellen Raum sollte durch geeignete Werkzeuge unterstützt werden. Das können sowohl Open-Source-Anwendungen zur Nutzung im Browser sein als auch proprietäre Anwendungen. Wichtig ist, dass in Echtzeit kommuniziert, kooperiert und reflektiert werden kann, damit die Teams optimale gemeinsam Lösungen erarbeiten können. Dabei sollten verschiedene Medientypen (Text, Audio, Bild, Video) integriert werden können, ohne dass die Teams von technischen Problemen abgelenkt werden. Bei unserer Challenge belief sich die IT-Unterstützung auf die Challenge-Plattform der *Xeem GmbH*. Unerfahrenen Organisationen, die sich für unternehmerische Innovationswettbewerbe interessieren, empfehlen wir, eine einfache Fragestellung in einem begrenzten Rahmen zu wählen und sich, wie in unserem Fall, externe Unterstützung zu holen. Experimentierfreudigere Organisationen, die ohne Hilfe eine virtuelle Challenge veranstalten wollen, können dies selbstständig mit Anwendungen für virtuelle Kollaboration (z. B. MIRO, Google Docs) und Kommunikation (z. B. ZOOM, Mattermost) tun.

#### Auswahl einer geeigneten methodischen Unterstützung (U2)

Die Teams sollten Methoden und Techniken zur Verfügung gestellt bekommen, die sie bei der Bearbeitung der Challenge-Fragestellung unterstützen. Für Design-Challenges eignen sich Techniken und Material zur Unterstützung des kollaborativen und kreativen Prozesses. In unserem Fall haben die Teams Anwendungshilfen zum Design-Thinking-Prozess und zu den Methoden Customer Journey Map, Persona und Denkhüte von De Bono erhalten. Die Dokumentation der Anwendung durch einige Teams ermöglichte es der Jury, neben der Lösung auch den Entstehungsprozess nachzuvollziehen.

## Fazit

Dieser Beitrag berichtet über die Durchführung einer virtuellen Design-Challenge zum Thema ‚Einkaufserlebnis der Zukunft in Innenstädten‘ und bietet praxisrelevante Empfehlungen zur Planung und Durchführung solcher Challenges. Ein Innovationsökosystem bestehend aus privaten und öffentlichen Organisationen setzt mit einem solchen Ideenwettbewerb ein Format um, das insbesondere auch offene Innovation ermöglicht. Die in diesem Beitrag verwendeten Frameworks und Konzepte können bei der Umsetzung eigener Innovationswettbewerbe genutzt werden. Wir hoffen, mit unserem Beitrag nützliche Impulse für den Einsatz von Formaten und Werkzeugen der offenen Innovation in privatwirtschaftlichen und öffentlichen Organisationen zu liefern. Auch wenn es sich um einen konkreten Innovationswettbewerb handelt, zeigen unsere Erfahrungen dennoch, dass solche Wettbewerbe zahlreiche Potenziale bieten. Für kleine und mittelständische Unternehmen mit begrenzten Ressourcen stellen zeitlich befristete Innovationsprojekte eine Chance dar. Dabei können sie in Zusammenarbeit mit anderen Organisationen neue Prozesse und Technologien erproben und so Vor- und Nachteile organisationaler Ambidextrie erkunden. Zudem stellen Innovationswettbewerbe ein innovatives Werkzeug zur Rekrutierung digital-affiner Fachkräfte dar. Außerdem können sie zur Weiterentwicklung der Kompetenzen der eigenen Belegschaft eingesetzt werden, wobei inbesondere die Kompetenzen des Lernens und Verlernens (z. B. hinderliche Routinen, Fiol und O’Connor 2017) unterstützt werden können. Auch Städte auf dem Weg zur Smart City können Innovationswettbewerbe einsetzen, um eine offene, inklusive und partizipative Gestaltung bürgernaher Städte zu ermöglichen und dabei die Vorteile der Digitalisierung zu nutzen (Leible et al. 2021). Universitäten können hierbei mit ihrem Wissen über Vorgehen und Technologien positiv unterstützen (Becker et al. 2020). Organisationen verschiedener Art können also von Maßnahmen der offenen Innovation profitieren (Hwang et al. 2021). Zusammenfassend lässt sich sagen, dass Design-Challenges im virtuellen Raum ein vielversprechendes Instrument für die Umsetzung offener Innovation darstellen, das sowohl von privatwirtschaftlichen als auch von öffentlichen Organisationen für vielfältige Zielsetzungen eingesetzt werden kann.

## Footnotes

1 www.digitaltag.eu/future-shopping-experience-virtual-challenge

2 www.pro-x-lab.de/

3 Hierfür wurde die Webanwendung ‚Mentimeter‘ verwendet (www.mentimeter.com).

4 Jedes Team wurde anhand von fünf Kriterien (Grad der Passung mit Challenge, Grad der Innovation, Grad der Ausarbeitung, Grad der Verständlichkeit, Grad der Nutzerfokussierung) auf einer 5‑fach-Skala (1: sehr gering; 5: sehr stark) von der Jury (Stadt Hildesheim, Compra/HiXDigiHub, ProXLab) bewertet. So konnte ein Team in Summe zwischen 15 und maximal 75 Punkten erreichen. Das Schema ist auf Anfrage erhältlich.
